# The first complete mitochondrial genome of marigold pest thrips, *Neohydatothrips samayunkur* (Sericothripinae) and comparative analysis

**DOI:** 10.1038/s41598-018-37889-6

**Published:** 2019-01-17

**Authors:** Vikas Kumar, Kaomud Tyagi, Shantanu Kundu, Rajasree Chakraborty, Devkant Singha, Kailash Chandra

**Affiliations:** Centre for DNA Taxonomy, Molecular Systematics Division, Zoological Survey of India, M- Block, New Alipore, Kolkata, 700 053 West Bengal India

## Abstract

Complete mitogenomes from the order Thysanoptera are limited to representatives of the subfamily Thripinae. Therefore, in the present study, we sequenced the mitochondrial genome of *Neohydatothrips samayunkur* (15,295 bp), a member of subfamily Sericothripinae. The genome possesses the canonical 13 protein-coding genes (PCGs), 22 transfer RNA genes (tRNAs), and two ribosomal RNA genes (rRNAs) as well as two putative control regions (CRs). The majority strand was 77.42% A + T content, and 22.58% G + C with weakly positive AT skew (0.04) and negative GC skew (−0.03). The majority of PCGs start with ATN codons as observed in other insect mitochondrial genomes. The GCG codon (Alanine) was not used in *N*. *samayunkur*. Most tRNAs have the typical cloverleaf secondary structure, however the DHU stem and loop were absent in *trnV* and *trnS1*, while the TΨC loop was absent in *trnR* and *trnT*. The two putative control regions (CR1 and CR2) show 99% sequence similarity indicated a possible duplication, and shared 57 bp repeats were identified. *N. samayunkur* showed extensive gene rearrangements, with 11 PCGs, 22 tRNAs, and two rRNAs translocated when compared to the ancestral insect. The gene *trnL2* was separated from the ‘*trnL2-cox2*’ gene block, which is a conserved, ancestral gene order found in all previously sequenced thrips mitogenomes. Both maximum likelihood (ML) and Bayesian inference (BI) phylogenetic trees resulted in similar topologies. The phylogenetic position of *N. samayunkur* indicates that subfamily Sericothripinae is sister to subfamily Thripinae. More molecular data from different taxonomic groups is needed to understand thrips phylogeny and evolution.

## Introduction

The order Thysanoptera (thrips) includes nine families in two suborders, the Terebrantia and Tubulifera. The family Thripidae is the largest of the Terebrantia and is further subdivided into four subfamilies; Dendrothripinae, Panchaetothripinae, Sericorthripinae, and Thripinae^1^. The members of Sericothripinae have a worldwide distribution and are usually associated with flowers^2,3^. This subfamily currently includes 168 species in three genera, *Neohydatothrips*, *Hydatothrips*, and *Sericothrips*. The marigold thrips, *Neohydatothrips samayunkur* is a pest of marigold (*Tagetes* spp.) with a worldwide distribution^4–6^. Recently, *N. samayunkur* has also been suspected as a vector for tospoviruses^7^. Integration of molecular data with morphology is required for fast and accurate species identification and to understand phylogenetic relationships^2^. The mitochondrial genes cox1 and 16S rRNA have been found to be useful in the identification of thrips species and to infer phylogenetic relationships^8–10^, however, phylogenetic relationships below the family level in thrips are still unclear and require more molecular data^2–4^.

Insects typically have a single circular mitochondrial genome, 14–19 kb in size, with 37 genes, including 13 protein-coding genes (PCGs), large and small ribosomal RNA genes (rRNAs), 22 transfer RNA genes (tRNAs) and variable number of A + T rich control regions (CRs). The characteristic features of the animal mitochondrial genomes are (i) conserved gene content, (ii) conserved genome size and organization, (iii) lack of extensive recombination, (iv) maternal inheritance, and (v) an accelerated rate of nucleotide substitution^11–13^. Therefore, this small molecule has been widely used in insect phylogenetic and evolutionary studies^14–16^.

To date, the highly rearranged mitogenomes of five thrips species (*Anaphothrips obscurus, Frankliniella intonsa, Frankliniella occidentalis, Scirtothrips dorsalis* and *Thrips imaginis*) are available^17–21^. However, the availability of thrips mitogenomes is limited to the subfamily Thripinae. In this study, we sequenced the complete mitochondrial genome of *N. samayunkur*, a member of subfamily Sericothripinae using next-generation sequencing (NGS) technology and compared it to other thrips mitogenomes, analysing genome organization, codon usage patterns, tRNA secondary structure and strand asymmetry. Phylogenetic relationships were inferred by analysing the 13 PCGs from published thrips mitogenomes using maximum likelihood (ML) and Bayesian inference (BI).

## Results and Discussion

### Genome structure, organization and composition

**Figure 1:**
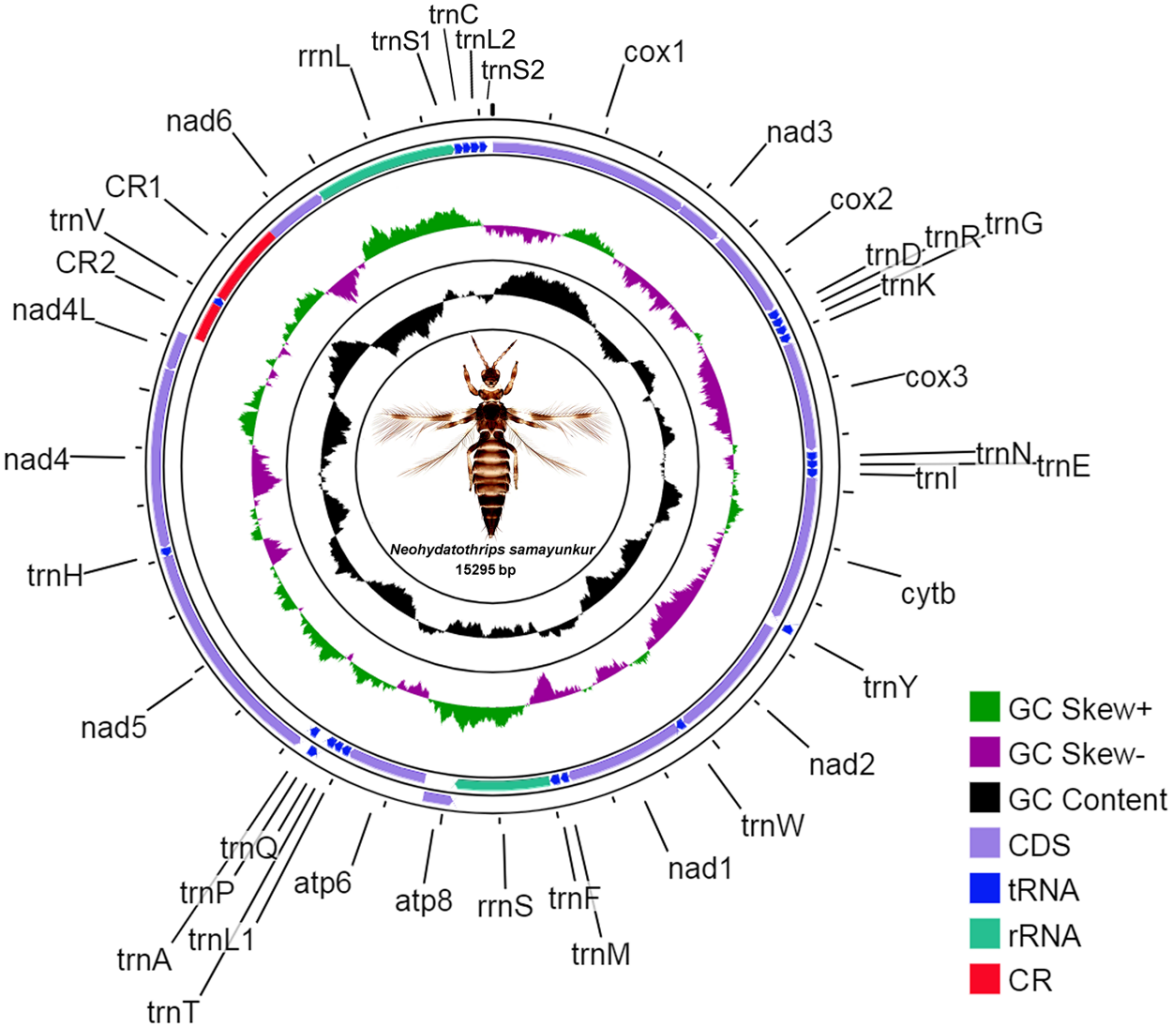
The mitochondrial genome of the marigold thrips, *N. samayunkur*. Direction of gene transcription is indicated by arrows in entire complete genome. PCGs are shown as purple arrows, rRNA genes as sea green arrows, tRNA genes as blue arrows and CR regions as red rectangles. The GC content is plotted using a black sliding window, as the deviation from the average GC content of the entire sequence. GC-skew is plotted using a colored sliding window (green and orchid color), as the deviation from the average GC-skew of the entire sequence. The figure was drawn using CGView online server (http://stothard.afns.ualberta.ca/cgview_server/) with default parameters. The species photograph was taken by the second author (KT) using Leica Microscope DM1000 with Leica software application suite (LAS EZ) and edited manually in Adobe Photoshop CS 8.0.

The complete sequence of the mitochondrial genome of *N*. *samayunkur* (accession number MF991901) is 15,295 base pairs (bp) in length. This is longer than those of *A. obscurus*, *F. intonsa*, *F. occidentalis*, and *S. dorsalis* South Asia (SA1), but smaller than the genomes of *T. imaginis* and *S. dorsalis* East Asia (EA1) (Table S1). As in other insect species, the mitochondrial genome of *N*. *samayunkur* included 37 genes: 13 PCGs, large and small rRNAs and 22 tRNAs but two putative CRs (Fig. 1). There are 204 bp intergenic nucleotides in total, across 23 locations, with individual spacer length of 1 to 41 bp. The longest intergenic spacer (41 bp) was located between the *trnS2* and *cox1* gene, with an extremely high AT content (85.37%). Three pairs of genes overlap with lengths ranging from 1 to 24 bp. Thirty genes are on the majority strand and seven on the minority (Table 1). Nucleotide composition was 77.42% A + T content and 22.58% G + C content (Table 2); similar to other thrips mitogenomes. A + T content was highest at 80.87%, in tRNAs, followed by rRNAs (79.31%), PCGs (77.15%), and CRs (71.12%). The mitogenome showed weakly positive AT (0.04) and negative GC (−0.03) skews (Table 2).

**Table 1:** List of annotated mitochondrial genes of *Neohydatothrips samayunkur* and its characteristic features.

Gene	Strand	Location	Size (bp)	Anti codon	Start codon	Stop codon	IGN
*cox1*	+	1–1536	1536	—	ATA	TAA	0
*nad3*	+	1537–1890	354	—	ATG	TAA	25
*cox2*	+	1916–2584	669	—	ATA	TAA	10
*trnD*	+	2595–2661	67	GAC	—	—	0
*trnR*	+	2662–2723	62	CGA	—	—	3
*trnG*	+	2727–2788	62	GGA	—	—	7
*trnK*	+	2796–2858	63	AAA	—	—	13
*cox3*	+	2872–3702	831	—	ATA	TAA	14
*trnN*	+	3717–3782	66	AAC	—	—	−3
*trnE*	+	3780–3842	63	GAA	—	—	0
*trnI*	+	3843–3909	67	ATC	—	—	4
*cytb*	+	3914–5014	1101	—	ATT	TAA	1
*trnY*	−	5016–5079	64	TAC	—	—	17
*nad2*	+	5097–6087	991	—	ATC	T(AA)	0
*trnW*	+	6088–6152	65	TGA	—	—	0
*nad1*	+	6153–7089	937	—	ATA	T(AA)	−24
*trnM*	+	7066–7126	61	ATG	—	—	8
*trnF*	+	7135–7206	72	TTC	—	—	6
*rrnS*	+	7213–7935	723	—	—	—	1
*atp8*	−	7937–8152	216	—	ATT	TAA	13
*atp6*	+	8166–8777	612	—	ATG	TAG	0
*trnL1*	+	8778–8841	64	CTA	—	—	5
*trnT*	+	8847–8906	60	ACA	—	—	−1
*trnQ*	+	8906–8974	69	CAA	—	—	10
*trnP*	−	8985–9050	66	CCA	—	—	8
*trnA*	+	9059–9121	63	GCA	—	—	10
*nad5*	−	9132–10817	1686	—	ATA	TAA	0
*trnH*	−	10818–10877	60	CAC	—	—	2
*nad4*	−	10880–12178	1299	—	ATT	TAA	2
*nad4L*	−	12181–12459	279	—	ATG	TAA	0
*CR2*	+	12460–12759	300	—	—	—	0
*trnV*	+	12760–12819	60	GTA	—	—	0
*CR1*	+	12820–13447	628	—	—	—	0
*nad6*	+	13448–13918	471	—	ATT	TAA	1
*rrnL*	+	13920–15004	1085	—	—	—	1
*trnS1*	+	15006–15069	64	TCA	—	—	0
*trnC*	+	15070–15129	60	TGC	—	—	0
*trnL2*	+	15130–15194	65	TTA	—	—	2
*trnS2*	+	15197–15254	58	AGA	—	—	0

The protein coding and ribosomal RNA genes are represented by standard nomenclature, tRNAs are represented as trn followed by the IUPAC-IUB single letter amino acid codes. (+) values in strand represent as heavy (H) and (−) values represent as light (L). IGN represents (+) values as intergenic nucleotides and (−) values as overlapping regions. CR represents the control region.

**Table 2:** Composition and skew in different Thysanoptera mitogenomes included for comparative analysis.

Species	Size(bp)	A%	G%	T%	C%	GC%	AT%	AT skew	GC skew
**Whole mtgenome**									
*N.samayunkur*	15,295	40.25	10.98	37.17	11.60	22.58	77.42	0.04	−0.03
*T. imaginis*	15,407	43.85	10.47	32.72	12.96	23.43	76.57	0.15	−0.11
*F. intonsa*	15,215	41.24	11.06	34.68	13.01	24.07	75.93	0.09	−0.08
*F. occidentalis*	14,889	40.98	11.35	36.62	11.06	22.41	77.59	0.06	0.01
*S. dorsalis* EA1	15,343	39.12	11.61	36.62	12.64	24.26	75.74	0.03	−0.04
*S. dorsalis* SA1	15,204	39.83	11.18	37.56	11.42	22.60	77.40	0.03	−0.01
*A. obscurus*	14,890	38.38	11.27	39.75	10.60	21.87	78.13	−0.02	0.03
**PCG**									
*N. samayunkur*	10,982	39.59	10.79	37.56	12.06	22.85	77.15	0.03	−0.06
*T. imaginis*	10,922	42.75	10.15	32.89	14.21	24.36	75.64	0.13	−0.17
*F. intonsa*	11,009	39.95	11.39	34.58	14.08	25.47	74.53	0.07	−0.11
*F. occidentalis*	10,852	39.82	11.62	36.72	11.84	23.46	76.54	0.04	−0.01
*S. dorsalis* EA1	10,954	38.06	11.92	36.53	13.48	25.41	74.59	0.02	−0.06
*S. dorsalis* SA1	10,973	38.94	11.36	37.67	12.03	23.38	76.62	0.02	−0.03
*A. obscurus*	11,167	37.36	11.46	39.93	11.25	22.71	77.29	−0.03	0.01
**tRNA**									
*N. samayunkur*	1,401	43.11	9.85	37.76	9.28	19.13	80.87	0.07	0.03
*T. imaginis*	1,492	43.83	9.45	36.66	10.05	19.50	80.50	0.09	−0.03
*F. intonsa*	1,392	43.53	10.70	35.78	9.99	20.69	79.31	0.10	0.03
*F. occidentalis*	1,380	42.39	10.58	37.39	9.64	20.22	79.78	0.06	0.05
*S. dorsalis* EA1	1,426	40.53	11.01	37.52	10.94	21.95	78.05	0.04	0.00
*S. dorsalis* SA1	1,429	41.36	10.43	38.21	10.01	20.43	79.57	0.04	0.02
*A. obscurus*	1,430	39.79	10.63	39.86	9.72	20.35	79.65	0.00	0.04
**rRNA**									
*N. samayunkur*	1,808	44.97	11.73	34.35	8.96	20.69	79.31	0.13	0.13
*T. imaginis*	1,876	47.65	10.77	32.14	9.43	20.20	79.80	0.19	0.07
*F. intonsa*	1,699	47.15	11.30	32.02	9.54	20.84	79.16	0.19	0.08
*F. occidentalis*	1,848	45.94	12.18	33.93	7.95	20.13	79.87	0.15	0.21
*S. dorsalis* EA1	1,775	43.21	11.89	34.99	9.92	21.80	78.20	0.11	0.09
*S. dorsalis* SA1	1,777	45.36	11.65	34.44	8.55	20.20	79.80	0.14	0.15
*A. obscurus*	1,812	43.16	11.70	36.59	8.55	20.25	79.75	0.08	0.16
**Control region**									
*N. samayunkur*	928	33.84	14.87	37.28	14.01	28.88	71.12	0.05	−0.03
*T. imaginis*	900	47.56	16.67	25.22	10.56	27.22	72.78	0.31	0.22
*F. intonsa*	942	41.72	7.86	38.22	12.21	20.06	79.94	0.04	−0.22
*F. occidentalis*	595	40.34	7.90	43.70	8.07	15.97	84.03	−0.04	−0.01
*S. dorsalis* EA1	1,775	43.21	11.89	34.99	9.92	21.80	78.20	0.11	0.09
*S. dorsalis* SA1	767	35.33	9.26	43.55	11.86	21.12	78.88	−0.10	−0.12
*A. obscurus*	145	25.52	8.97	62.76	2.76	11.72	88.28	−0.42	0.53

### Protein-coding genes

All 13 PCGs used ATN start codons (five with ATA, four with ATT, three with ATG and one with ATC) as is observed in most of the insect mitochondrial genomes^22,23^. The stop codon TAA was used by 10 PCGs, and TAG for *atp6*, while an incomplete stop codon is present in *nad1* and *nad2*. Comparative analysis of start and stop codons among thrips showed the unique features of *N*. *samayunkur*: ATT start codon in *cytb* and *nad6*, ATC in *nad2*, and ATG in *atp6*. The complete stop codon TAA was used by *atp8* in *N. samayunkur*, while it was terminated by an incomplete stop codon T(AA) in other thrips species (S2 Table). The detection of an incomplete stop codon in *atp8* gene may be due to misannotation, as *atp8*-*atp6* is a conserved ancestral gene block with no tRNA between them^24^.

The entire length of PCGs of *N. samayunkur* was 10,982 bp. Overall A + T content of 13 PCGs was 77.15% in *N. samayunkur*, while it ranges from 74.53% to 77.29% across thrips. Codon usage in *N. samayunkur* shows a significant bias towards A/T rich codons. Relative synonymous codon usage analysis of *N*. *samayunkur* revealed that the codon GCG (Alanine) was not present at all. The most frequently utilized amino acids were Lysine (K), Phenylalanine (F), Leucine (L), Isoleucine (I), Tyrosine (Y), and Serine (S) as in other insects (S3 Table).

### Ribosomal and transfer RNA genes

*N*. *samayunkur* has two rRNAs as in other insects. The large ribosomal gene (16S) was 1085 bp long, and located between *nad6* and *trnS1*; the small (12S) was 723 bp long, located between *trnF* and *atp8* (Table 2). A + T content of two rRNAs was 79.31%, while it ranges from 79.16% (*S. dorsalis* EA1) to 79.87% (*F. occidentalis*) observed in other thrips.

*N. samayunkur* contained a complete set of 22 tRNAs (total length 1,401 bp) individually ranging from 58 to 72 bp in length. Collectively tRNAs have the highest A + T content 80.87% of any gene group (78.05% to 80.87% in thrips) (Table 2). Most tRNAs have the typical cloverleaf secondary structure except *trnV*, *trnS1*, *trnR*, and *trnT*. The DHU stem and loop were absent in *trnV* and *trnS1* while TΨ C loop absent in *trnR* and *trnT* (Fig. S1). The absence of DHU stem and loop in *trnV* is consistent across all thrips species sequenced to date.

### Control regions

**Figure 2:**
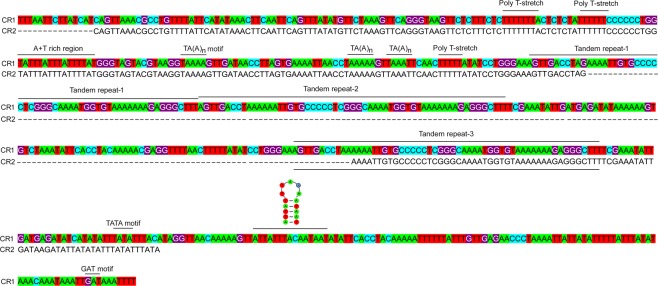
Comparison of the nucleotide sequences of two putative control regions of *N. samayunkur*. Four types of sequences were recognized in the control regions: tandem repeats, Poly T-stretches, A + T-rich sequences, TA(A)n motif, TATA motif, GAT motif and stem and loop. The figure was edited in Adobe Photoshop CS 8.0.

Control regions (CRs) in mitogenomes play an important role in transcription and replication^25^. A CR was found with following conserved elements; a poly T stretch at the 5′ end, a TA(A)n-like stretch, a stem and loop structure, a TATA motif, and a GAT motif^26–28^. The *N. samayunkur* mitogenome contains two putative control regions, CR1 (628 bp) and CR2 (300 bp), located between *trnV* and *nad6*, and *trnV* and *nad4L* respectively. CR2 had 99% sequence similarity with CR1, indicating a possible duplication. Three near tandem repeats (57 bp) were identified in CR1, while one repeat sequence was present in CR2 (Fig. 2). Most thrips species have been documented to have multiple CRs except *A. obscurus*. Three CRs are present in *F. intonsa*, *F. occidentalis*, and *S. dorsalis* SA1, two in *T. imaginis*, *S. dorsalis* EA1, and one in *A. obscurus*^18–21^. A location of CR1 upstream of *nad5* gene has been suggested to be ancestral condition of thrips^17^, however, the CR locations in *N. samayunkur* (subfamily Sericothripinae) differ from those of other thrips.

### Gene arrangement

**Figure 3:**
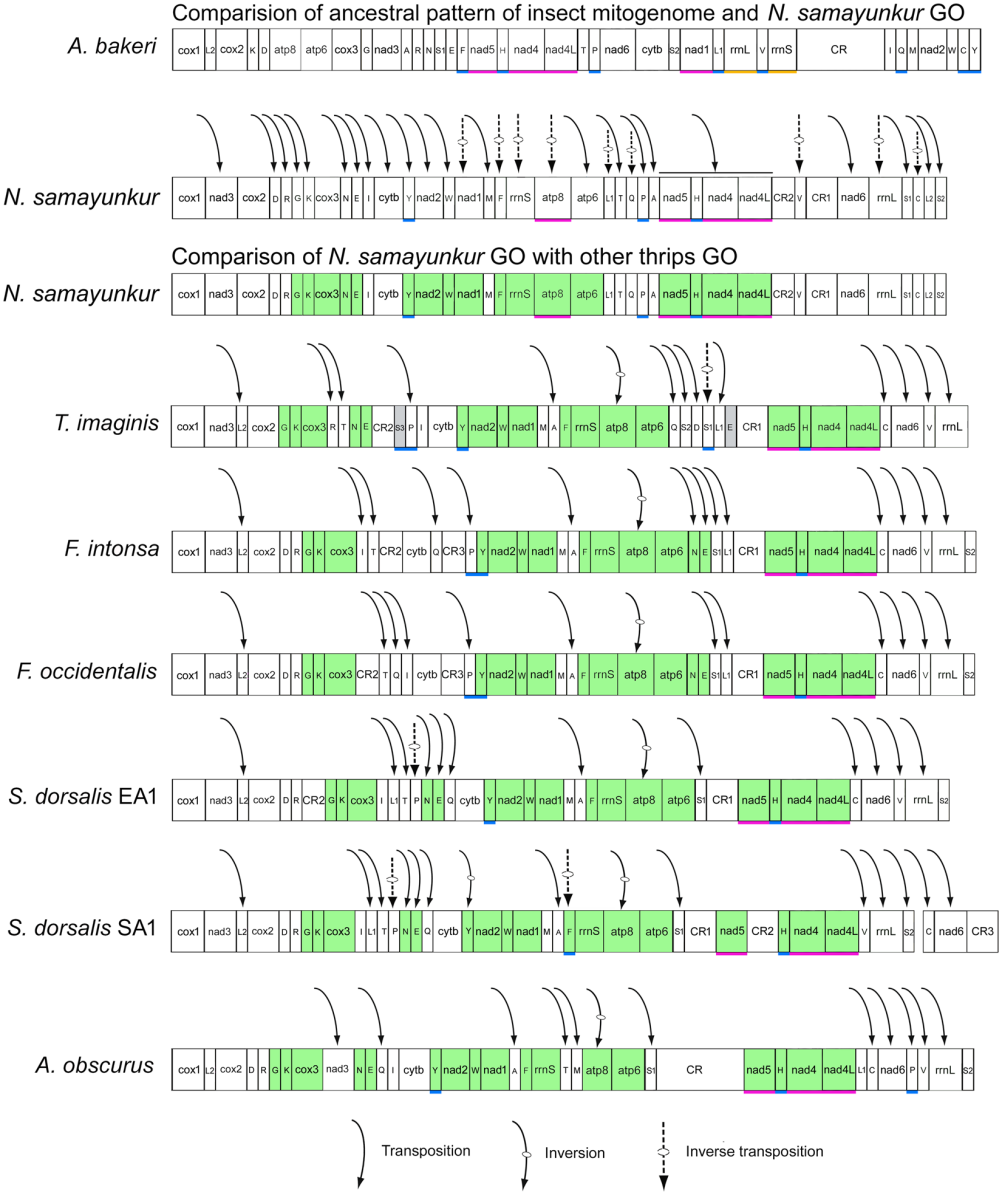
Linearized view of complete mitochondrial genome organization and gene rearrangement, transposition, inversion, and inverse transposition in *N. samayunkur* compared with the ancestral insect gene order. The green color blocks show the conserve gene blocks. The grey color blocks showed the pseudo genes of *T. imaginis*. Genes nomenclature: *atp6* and *atp8*; ATP synthase subunits 6 and 8; *cytb*: cytochrome b; *cox1*–*3*: cytochrome c oxidase subunits 1–3; *nad1*–*6* and *nad4L*: NADH dehydrogenase subunits *1*–*6* and *4L*; *rrnS* and *rrnL*: small and large subunit ribosomal RNA (rRNA) genes; Transfer RNA genes are denoted by a one-letter symbol according to the IPUCIUB single-letter amino acid codes. CR indicates the control regions. The figure was edited in Adobe Photoshop CS 8.0.

The mitogenome gene arrangements have been characterized by following patterns, transpositions, inversions, and inverse transpositions^11,29,30^. Tandem duplication–random loss (TDRL) is the most widely accepted process to explain transpositions^11^. The gene arrangement of *N. samayunkur* was assessed by comparing the common intervals with the ancestral insect gene order as an outgroup^17,31^. CREx^32^ analysis identified eight gene rearrangement events in *N. samayunkur*, including four inversions plus four TDRLs, assignable to two sets of alternative scenarios (Fig. S2). CREx detected inversions of *atp8*, *trnF*, *trnC* and gene block *nad1*-*rrnS* in both scenarios. *N. samayunkur* is a highly rearranged mitogenome with rearrangements of 11 PCGs, 22tRNAs, and two rRNAs as compared with the ancestral insect (Fig. 3). The majority of rearrangements were transpositions, while nine rearrangements (*nad1*, *atp8*, *trnF*, *trnL1*, *trnQ*, *trnV*, *trnC*, *rrnS*, and *rrnL*) were inverse transpositions. Further, when *N. samayunkur* was compared to other thrips species, the following derived gene blocks: *trnG*-*cox*3, *trnN*-*trnE*, *trnY*-*nad1*, *trnF*-*atp*6, and *nad5*-*nad4L* were conserved in all thrips species. Within the conserved gene block *trnF*-*atp6*, *atp8* was subsequently inverted in *N. samayunkur*. The following tRNAs were inverted in thrips species as compared to the ancestral insect: *trnY* in *S. dorsalis* SA1, *trnP* in both *S. dorsalis*, *trnS*1 in *T. imaginis*, and *trnF* in all species except *S. dorsalis* SA1. The gene *trnL2* was transposed in *N. samayunkur* away from the gene block *trnL2*-*cox2*, which is conserved in most insects including thrips (Fig. 3). The gene block *trnD*-*cox3* is conserved in five thrips species including *N. samayunkur*, while *trnD* and *trnR* were translocated in *T. imaginis* and interrupted by the CR2 in *S*. *dorsalis* EA1. The gene block *nad4L*-*nad5* is ancestral in insects and conserved in all thrips species. The conserved gene blocks *trnY*-*nad1* and *atp*6-*trnF* are separated by *trnM* and *trnA* in most of the thrips species except *N. samayunkur* (*trnM* alone) and *A. obscurus* (*trnA* alone).

### Strand asymmetry

AT and GC skews on the majority strand are used to measure strand asymmetry^33–35^. Most insects have positive AT skew (A > T) and negative GC skew (C > G). A reversal of the strand asymmetry (T > A and G > C) has been observed in a few species, and is proposed to be caused by the inversion of the replication origin within the control region^26–28,35^. *N. samayunkur* showed weakly positive AT skew (0.04) and negative GC skew (−0.03), similar to most other thrips species (Table 2). AT skew in other thrips species, ranges from −0.02 (*A*. *obscurus*) to 0.15 (*T*. *imaginis*), while GC skew varies from −0.11 (*T*. *imaginis*) to 0.03 (*A*. *obscurus*). Insect species with reversal of strand asymmetry have faster rate of gene rearrangements, however, species with faster rate of gene rearrangements do not always show reversal of strand asymmetry^35^. Two species of thrips (*A. obscurus* and *F. occidentalis*) showed weekly positive GC skew value. However, there is no inversion of replication related elements in CR indicating that strand asymmetry is not reversed in thrips.

### Phylogenetic analysis

**Figure 4:**
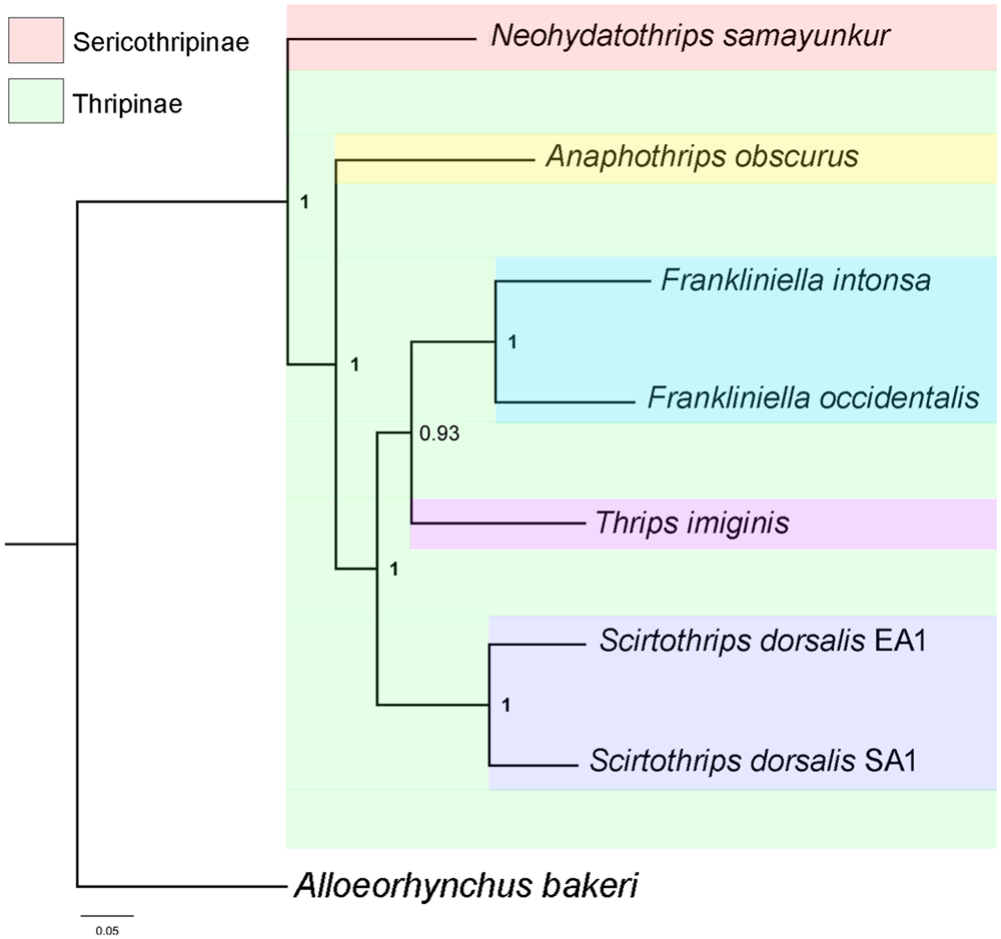
Phylogenetic tree inferred from nucleotide sequences of 13 PCGs using Bayesian Inference method in MrBayes v3.2. The tree is drawn to scale with bayesian posterior probability values indicated along with the branches. The figure was edited in Adobe Photoshop CS 8.0.

**Figure 5:**
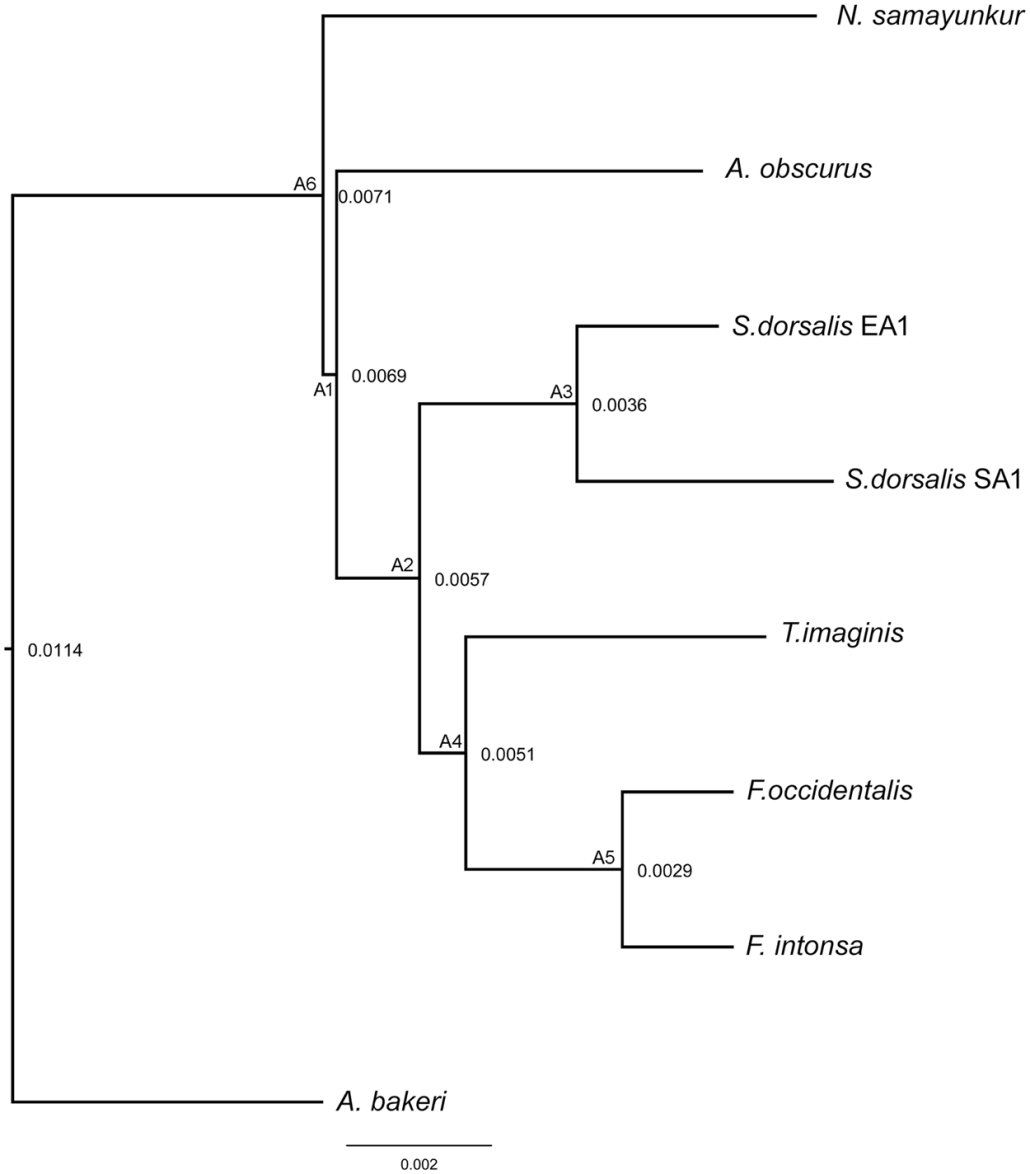
Phylogenetic tree inferred from MLGO web server based on gene arrangements.

Both Maximum likelihood (ML) and Bayesian Inference (BI) phylogenetic trees resulted in similar topologies (Fig. S3, Fig. 4). BI posterior probabilities (PP) were higher than ML bootstrap support (BS) values. It has been suggested that PP and BS values are not directly comparable and interchangeable, as PP gives higher nodal support than BS^36^. Species within the same genus, *F. intonsa* and *F. occidentalis* were grouped together and closely related to *T. imaginis*. The two cryptic species of *S. dorsalis* (EA1 and SA1) also clustered together and were closely related to the *Frankliniella* + *Thrips* clade. The four genera in the subfamily Thripinae: *Anaphothrips*, *Frankliniella*, *Scirtothrips*, and *Thrips* grouped together. *N. samayunkur* shows a sister relatioship to the Thripinae clade in the present phylogeny. Although, gene order is extensively rearranged among thrips mitogenomes, the branching pattern inferred by MLGO^37^ is congruent with the PCGs based ML and BI phylogeny (Fig. 5). Previous studies showed a close relationship between *T. imaginis* with *S. dorsalis*^17^, however, our study found that *T. imaginis* was closer to *Frankliniella* than to *Scirtothrips*, congruent with morphological understanding of these taxa^38^.

Previous studies described a close relationships between *Scirtothrips* (subfamily Thripinae) and *Neohydatothrips* (subfamily Sericothripinae) using both morphological^6^ and molecular data^8,9^. These two genera share the following morphological characters:^6^ presence of closely spaced rows of microtrichia on lateral thirds of abdominal tergites; median pair of tergal setae close together; campaniform sensilla absent on tergite IX, tergite X not split longitudinally. The suborder Terebrentia is typically classified into eight families with four subfamilies^39^; however, an alternative view proposes 28 families based on highly conserved taxonomic characters and elevates the subfamily Sericothripinae to family rank^40^. According to Bhatti, the proposed family Sericothripidae can be separated from other families of suborder Terebrantia by the presence of sublateral callosities on antecostal line on tergites II to VII and sternites II to VI (II to VIII in male), prominent anteriorly directed sublateral apodeme on each side on female sternites VII, one pair of cervical sclerites, annular rows of microtrichia on femora and tibiae, short and straight hind coxal apodeme, metathoracic furca not elongate and lyre-shaped, metasternellum somewhat to strongly enlarged, forming a transversely striate area on each side of the mid line, mesothorax with sternal coxal process small and inconspicuous; trochantin small and inconspicuous, absence of sclerites anterior to mesoacrotergite, one anteriorly directed apodeme on each side of sternite I. Moreover, previous studies clearly stated that the relationships between Thripinae (1779 species in 234 genera) and Sericothripinae (168 species in 3 genera) were unclear due to the absence of molecular data^8,41^. The present phylogenetic analysis contradicts a close relationship between *Scirtothrips* and *Neohydatothrips*.

The mitogenomes of two cryptic species of *S. dorsalis* (SA1 and EA1) vary considerably with respect to gene rearrangement and chromosome size^20^. *S. dorsalis* was described from “castor and chillies” at Coimbatore, India^42^. It is a polyphagous pest and a vector of tospoviruses with a global distribution. Earlier studies indicated that this species is a complex, consisting of many morphologically indistinguishable species^43,44^. Recently, nine cryptic species of *S. dorsalis* were delimited using multilocus molecular data^45^, however, these cryptic species has never been morphologically treated to validate and describe these species. Tagging specimens with the correct species name is a major problem as it is difficult to ascertain which of these cryptic species represent the true *S. dorsalis*.

To date, molecular phylogenetic studies of thrips is in its early stages due to lack of large scale data and taxonomic sampling.The generation of comprehensive molecular data on families/subfamilies is still needed.

## Materials and Methods

### Sample collection and DNA extraction

Adult specimens of *N. samayunkur* were collected from Odisha State, India. The studied species are common pests of crops, thus no prior permission was required for collection. Specimens were morphologically identified by the second author (K.T.) with available taxonomic keys^2,10^, and preserved in absolute ethyl alcohol at −30 °C in Centre for DNA Taxonomy, Molecular Systematics Division, Zoological Survey of India, Kolkata. Genomic DNA was extracted using DNeasy (QIAGEN) following the manufacturer’s standard protocol. Concentration of DNA was determined using a Qubit fluorometer with a dsDNA high-sensitivity kit (Invitrogen), and by agarose gel (0.8%) electrophoresis.

### Mitogenome sequencing and assembly

The whole genome library of genomic DNA was sequenced using the Illumina Hiseq2500 (2 × 150 base paired-end reads) (Illumina, USA) platform which yielded ~23 million reads. The paired-end library was constructed according to standard protocols for the TruSeq DNA Library Preparation kit (https://support.illumina.com/downloads/truseq). Raw sequencing reads were trimmed and quality filtered using the NGS-Toolkit^46^ to removing adapter contamination and low-quality reads (N’s or more than 70% of bases with a quality score < 20). High quality reads were filtered by using the Burrows-Wheeler Alignment (BWA) tool^47^ and assembled with SPAdes 3.9.0^48^, using default parameters, and the *S. dorsalis* mitochondrial genome (NC_025241.1) as a reference. Aligned reads were used for de novo mitochondrial genome assembly.

### Genome annotation, visualization, and comparative analysis

The assembled mitogenome was annotated using the MITOS web-server (http://mitos.bioinf.uni-leipzig.de/index.py)^49^. PCGs and rRNAs were confirmed manually by BLASTn, BLASTp and ORF Finder in NCBI^50,51^ (https://www.ncbi.nlm.nih.gov/orffinder/). Nucleotide sequences from protein coding genes (PCGs) were translated into putative proteins on the basis of the invertebrate mitochondrial genetic code. Initiation and termination codons were identified in ClustalX^52^ using other thrips reference mitogenome sequences. MEGA6^53^ was used for the alignment of homologous sequences across thrips species. The complete annotated mitogenome was submitted to NCBI GenBank using Sequin tool (http://www.ncbi.nlm.nih.gov/Sequin/). The circular map of the *N. samayunkur* mitogenome was illustrated by the CGView online server (http://stothard.afns.ualberta.ca/cgview_server/) with default parameters^54^. MEGA6 was used for estimation of nucleotide composition, codon usages, relative synonymous codon usage (RSCU) and composition of skewness with the following formula: AT skew = (A − T)/(A + T) and GC skew = (G − C)/(G + C)^55^. Secondary structures of transfer RNA (tRNA) genes were predicted by MITOS and further confirmed using tRNAscan-SE (http://lowelab.ucsc.edu/tRNAscan-SE/)^56^ and ARWEN 1.2^57^. RNAstructure version 6.0.1 was used to predict possible secondary structure within CRs^58^. Homology between CR1 and CR2 in *N. samayunkur* was determined through the ClustalW sequence alignment tool implemented in MEGA6. Gene arrangements pathways in *N. samayunkur* were evaluated by CREx (Common Interval Rearrangement Explorer)^32^.

### Phylogenetic analysis

Six complete mitogenomes of five thrips species were retrieved from GenBank on 1^st^ November 2017 for phylogenetic inference (S1 Table). The *A. bakeri* mitogenome was used as an out group^31^. Each PCG was aligned individually using the MAFFT algorithm in the TranslatorX^59^ online platform under the L-INS-i strategy based on codon-based multiple alignment. Poorly aligned nucleotides (1652 bp) were removed from the protein alignment using GBlocks (within TranslatorX) with default settings. The resulting alignments were concatenated by using Sequence Matrix1.7.8^60^. Concatenated dataset (9330 bp) was used for Bayesian inference (BI) and maximum likelihood (ML) analysis. PartitionFinder version 2.1.1^61^, with the greedy algorithm was used to find the best substitution models and partition schemes. Partitions were predefined for the codon positions for each PCGs (13 genes X 3 codons = 39 partitions). The BI analysis was performed using Mr. Bayes 3.2^62^ with HKY + I + G, TVM + G, TRN + G, GTR + I + G, HKY + G, GTR + I, TVM + I + G model estimated by PartitionFinder (S4 Table). Two runs each with four chains (three heated and one cold) for 500,000 generations, and trees were sampled every 100 generations. A consensus tree was acquired and visualized after excluding the first 25% trees as burn-in. The ML analysis was performed using the IQ-TREE^63^ Web Server in W-IQ-TREE^64^ (http://iqtree.cibiv.univie.ac.at/) with 1,000 replicates of ultrafast likelihood bootstrap^65^. The phylogenetic tree was visualized and edited using FigTree v1.4.2 (http://tree.bio.ed.ac.uk/software/figtree/)^66^. Phylogenetic relationships of studied taxa were also estimated based on gene arrangement patterns in the MLGO web server^37^ (S5 Table).

## Supplementary information

Supplementary info
