# Supplementary material for: The first complete mitochondrial genome of marigold pest thrips, Neohydatothrips samayunkur (Sericothripinae) and comparative analysis
Source: Sci Rep. 2019 Jan 17;9:191. doi: 10.1038/s41598-018-37889-6 (PMC6336932; doi:10.1038/s41598-018-37889-6)
Supplement: Supplementary file 1 — Supplementary info [file 41598_2018_37889_MOESM1_ESM.docx]

**Supplementary Information**

**The first complete mitochondrial genome of marigold pest thrips, *Neohydatothrips samayunkur* (Sericothripinae) and comparative analysis**

Vikas Kumar, Kaomud Tyagi***, Shantanu Kundu, Rajasree Chakraborty, Devkant Singha, Kailash Chandra

*Centre for DNA Taxonomy*, *Molecular Systematics Division*, *Zoological Survey of India*, *M- Block*, *New Alipore*, *Kolkata- 700 053*, *West Bengal*, *India*

**Corresponding author’s Email: kumud.tyagi5@gmail.com*

**Figure S1.
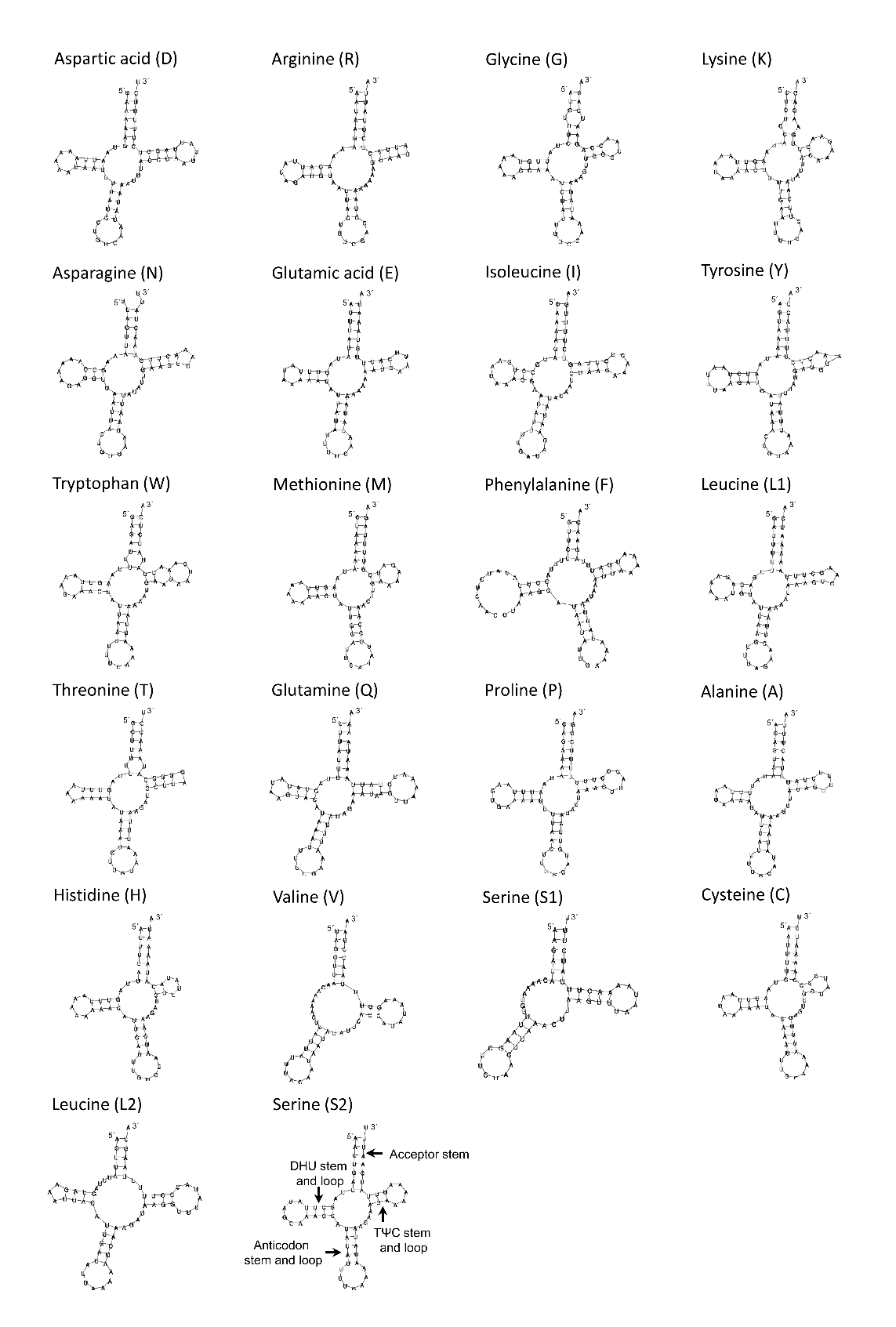
Putative secondary structures of the 22 tRNA genes of the *N. samayunkur* mitogenome.** The tRNAs are represented by full names and IUPAC-IUB single letter amino acid codes. The details of stem and loop is mentioned for one tRNA Serine (S2) which is applicable for all tRNAs secondary structures. The secondary structure of tRNAs were predicted by MITOS online server (http://mitos.bioinf.uni-leipzig.de/index.py) and edited manually in Adobe Photoshop CS 8.0.

**
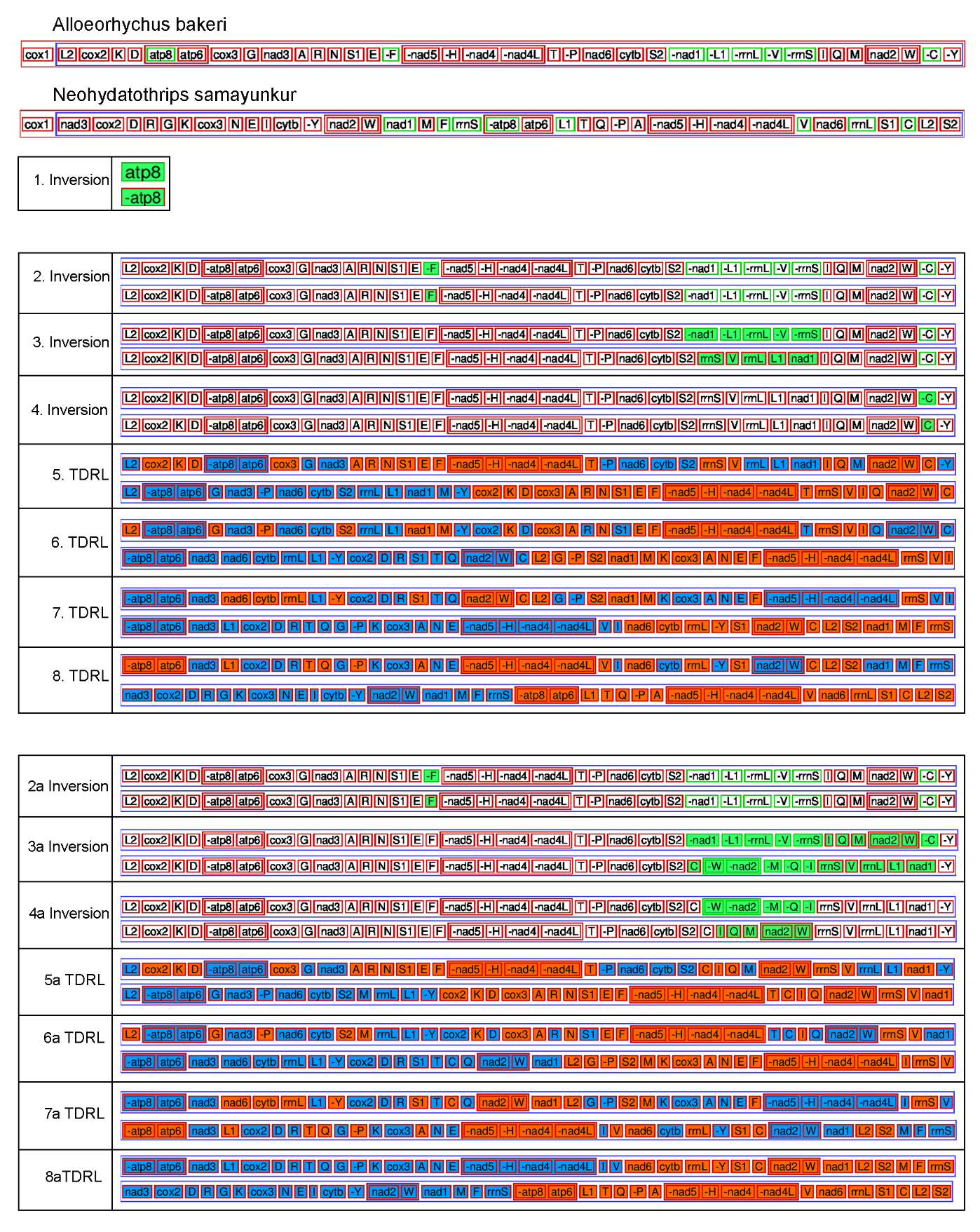
Figure S2. Evolution of gene order in mitochondrial genome of *N. samayunkur* explained by CREx.** In total eight rearrangement operations occurred from presumed ancestral gene order of *A. bakeri* to form the derived gene order of *N. samayunkur* gene order. Two alternative sets of scenarios were found, i.e. operations 1–8 and operations 2a–8a.

**Figure S3. Maximum Likelihood** Phylogenetic tree inferred from nucleotide sequences of 13 PCGs using IQ-TREE Web Server in W-IQ-TREE (<http://iqtree.cibiv.univie.ac.at/>) with 1,000 replicates of ultrafast likelihood bootstrap. The tree is drawn to scale with bootstrap values indicated along with the branches.

**Table S1. Details of the Thysanoptera mitogenomes generated till date and considered for comparative mitogenome study.**

| **Subfamily** | **Species** | **Accession No.** | **Size (bp)** | **GC%** | **PCG** | **rRNA** | **tRNA** | **CR** |
| --- | --- | --- | --- | --- | --- | --- | --- | --- |
| [Sericothripinae](https://www.ncbi.nlm.nih.gov/Taxonomy/Browser/wwwtax.cgi?mode=Undef&id=333393&lvl=3&keep=1&srchmode=1&unlock) | *Neohydatothrips samayunkur* | MF991901 | 15,295 | 22.6 | 13 | 2 | 22 | 2 |
| Thripinae | *Anaphothrips obscurus* | KY498001 | 14,890 | 21.87 | 12 | 2 | 22 | 1 |
| Thripinae | *Scirtothrips dorsalis* EA1 | KM349826 | 15,343 | 24.26 | 13 | 2 | 22 | 2 |
| Thripinae | *Scirtothrips dorsalis* SA1 | KM349827 & KM349828 | 15,204 | 22.6 | 13 | 2 | 22 | 3 |
| Thripinae | *Frankliniella intonsa* | JQ917403 | 15,215 | 24.07 | 13 | 2 | 22 | 3 |
| Thripinae | *Frankliniella occidentalis* | JN835456 | 14,889 | 22.41 | 13 | 2 | 22 | 3 |
| Thripinae | *Thrips imaginis* | AF335993 | 15,407 | 23.43 | 13 | 2 | 23 | 2 |

**Table S2. Comparison of the start and stop codons of the PCGs of the thrips species.**

| **PCGs** | ***N. samayunkur*** | | ***A. obscurus*** | | ***S. dorsalis* EA1** | | ***S. dorsalis* SA1** | | ***F. intonsa*** | | ***F. occidentalis*** | | ***T. imaginis*** | |
| --- | --- | --- | --- | --- | --- | --- | --- | --- | --- | --- | --- | --- | --- | --- |
|  | **Start** | **Stop** | **Start** | **Stop** | **Start** | **Stop** | **Start** | **Stop** | **Start** | **Stop** | **Start** | **Stop** | **Start** | **Stop** |
| *cox1* | ATA | TAA | ATC | TAG | ATG | TAA | ATG | TAA | ATG | TAA | ATG | TAA | ATA | TAA |
| *nad3* | ATG | TAA | ATA | TAA | ATT | TAA | ATT | TAA | ATA | TAA | ATT | TAA | ATT | TAA |
| *cox2* | ATA | TAA | ATA | TAA | TTG | TAG | TTG | TAG | ATT | TAA | ATA | TAA | ATA | TAA |
| *cox3* | ATA | TAA | ATA | TAA | ATA | TAA | ATA | TAA | ATT | TAA | ATT | TAG | ATA | TAA |
| *cytb* | ATT | TAA | ATA | TAA | ATA | TAG | ATA | TAA | ATA | TAA | ATA | TAA | ATA | TAA |
| *nad2* | ATC | T(AA) | ATA | TAA | ATA | T(AA) | ATA | T(AA) | ATA | T(AA) | ATA | TAA | ATA | T(AA) |
| *nad1* | ATA | T(AA) | ATA | TAA | ATA | TAA | ATA | TAA | ATA | T(AA) | ATT | T(AA) | ATA | T(AA) |
| *atp8* | ATT | TAA | ATT | T(AA) | ATG | T(AA) | ATG | T(AA) | ATG | T(AA) | ATT | T(AA) | ATA | T(AA) |
| *atp6* | ATG | TAG | ATA | TAA | ATT | TAA | ATA | TAA | ATT | TAA | ATT | TAA | ATT | TAA |
| *nad5* | ATA | TAA | ATA | TAA | ATA | TAG | ATA | TAG | ATT | TAG | ATT | TAA | ATT | T(AA) |
| *nad4* | ATT | TAA | ATA | TAA | ATT | T(AA) | ATT | T(AA) | ATT | T(AA) | ATT | T(AA) | TAAA | T(AA) |
| *nad4L* | ATG | TAA | ATG | TAA | ATT | T(AA) | ATT | TAA | ATG | T(AA) | ATA | T(AA) | ATT | TAG |
| *nad6* | ATT | TAA | ATA | TAA | ATA | TAA | ATA | TAA | ATA | TAA | ATA | TAA | ATA | TAA |

**Table S3. RSCU analysis of the PCGs of in *N. samayunkur* mitogenome.**

| **Amino acid** | **Codon** | **Number** | **Frequency (%)** | **RSCU** | **Amino acid** | **Codon** | **Number** | **Frequency (%)** | **RSCU** |
| --- | --- | --- | --- | --- | --- | --- | --- | --- | --- |
| Phenylalanine | UUU | 315 | 8.61 | 1.62 | Tyrosine | UAU | 192 | 5.25 | 1.54 |
|  | UUC | 75 | 2.05 | 0.38 |  | UAC | 58 | 1.58 | 0.46 |
| Leucine | UUA | 147 | 4.02 | 2.62 | **Stop codon*** | UAA | 133 | 3.63 | 1.53 |
|  | UUG | 40 | 1.09 | 0.71 |  | UAG | 41 | 1.12 | 0.47 |
|  | CUU | 68 | 1.86 | 1.21 | Histidine | CAU | 40 | 1.09 | 1.43 |
|  | CUC | 23 | 0.63 | 0.41 |  | CAC | 16 | 0.44 | 0.57 |
|  | CUA | 46 | 1.26 | 0.82 | Glutamine | CAA | 52 | 1.42 | 1.82 |
|  | CUG | 13 | 0.36 | 0.23 |  | CAG | 5 | 0.14 | 0.18 |
| Isoleucine | AUU | 232 | 6.34 | 1.66 | Asparagine | AAU | 267 | 7.30 | 1.6 |
|  | AUC | 48 | 1.31 | 0.34 |  | AAC | 67 | 1.83 | 0.4 |
| Methionine | AUA | 145 | 3.96 | 1.58 | Lysine | AAA | 335 | 9.15 | 1.68 |
|  | AUG | 38 | 1.04 | 0.42 |  | AAG | 65 | 1.78 | 0.33 |
| Valine | GUU | 39 | 1.07 | 2.03 | Aspartic acid | GAU | 54 | 1.48 | 1.61 |
|  | GUC | 3 | 0.08 | 0.16 |  | GAC | 13 | 0.36 | 0.39 |
|  | GUA | 27 | 0.74 | 1.4 | Glutamic acid | GAA | 86 | 2.35 | 1.85 |
|  | GUG | 8 | 0.22 | 0.42 |  | GAG | 7 | 0.19 | 0.15 |
| Serine | UCU | 64 | 1.75 | 1.29 | Cysteine | UGU | 66 | 1.80 | 1.43 |
|  | UCC | 37 | 1.01 | 0.75 |  | UGC | 26 | 0.71 | 0.57 |
|  | UCA | 55 | 1.50 | 1.11 | Tryptophan | UGA | 52 | 1.42 | 1.17 |
|  | UCG | 9 | 0.25 | 0.18 |  | UGG | 37 | 1.01 | 0.83 |
| Proline | CCU | 40 | 1.09 | 1.6 | Arginine | CGU | 2 | 0.05 | 0.29 |
|  | CCC | 17 | 0.46 | 0.68 |  | CGC | 8 | 0.22 | 1.14 |
|  | CCA | 40 | 1.09 | 1.6 |  | CGA | 14 | 0.38 | 2 |
|  | CCG | 3 | 0.08 | 0.12 |  | CGG | 4 | 0.11 | 0.57 |
| Threonine | ACU | 50 | 1.37 | 1.27 | Serine | AGU | 49 | 1.34 | 0.99 |
|  | ACC | 38 | 1.04 | 0.96 |  | AGC | 31 | 0.85 | 0.63 |
|  | ACA | 54 | 1.48 | 1.37 |  | AGA | 91 | 2.49 | 1.84 |
|  | ACG | 16 | 0.44 | 0.41 |  | AGG | 60 | 1.64 | 1.21 |
| Alanine | GCU | 19 | 0.52 | 1.69 | Glycine | GGU | 14 | 0.38 | 1.04 |
|  | GCC | 4 | 0.11 | 0.36 |  | GGC | 1 | 0.03 | 0.07 |
|  | GCA | 22 | 0.60 | 1.96 |  | GGA | 30 | 0.82 | 2.22 |
|  | GCG | 0 | 0.00 | 0 |  | GGG | 9 | 0.25 | 0.67 |

**Table S4.** **The best schemes of partition and substitution models used for each partition optimal partition Model Initial partition.**

| **Partitions** | **Model** | **PCGs-codon positions** |
| --- | --- | --- |
| Partition 1 | HKY+I+G | nad6-2, nad2-1, atp8-3, atp8-1 |
| Partition 2 | TVM+G | cox2-2, nad4L-2, nad2-2, nad6-3, nad5-1, nad4-3, cox3-1, nad1-2, nad3-1, atp6-2 |
| Partition 3 | TRN+G | atp8-2, nad1-3, nad6-1, nad2-3, atp6-3 |
| Partition 4 | GTR+I+G | nad3-3, nad1-1, atp6-1, cox3-3, cox2-1, cytb-2 |
| Partition 5 | HKY+G | cox1-1, nad3-2, cytb-1, cox2-3, cox3-2 |
| Partition 6 | GTR+I | cox2-2 |
| Partition 7 | TVM+I+G | cytb-3, cox1-3 |
| Partition 8 | HKY+G | nad4L-3, nad5-2, nad4-1 |
| Partition 9 | GTR+I+G | nad4L-1, nad4-2, nad5-3 |

**Table S5.** **Input file for MLGO analysis and phylogenetic relationships within Thysanoptera using gene order.** Input data (genes are as in Ancestral pattern of insect mitochondrial gene arrangement of Figure 3).

| > *Alloeorhynchus bakeri* (AB)  1 2 3 4 5 6 7 8 9 10 11 12 13 14 15 -16 -17 -18 -19 -20 21 -22 23 24 25 -26 -27 -28 -29 -30 31 32 33 34 35 36 -37 -38 $  >*Neohydatothrips samayunkur* (NS)  1 10 3 5 12 9 4 8 13 15 32 24 -38 35 36 26 34 16 30 -6 7 27 21 33 -22 11 -17 -18 -19 -20 39 29 31 23 28 14 37 2 25 $  >*Anaphothrips obscurus* (AO)  1 2 3 5 12 9 4 8 10 13 15 33 32 24 -38 35 36 26 11 16 30 21 34 6 7 14 31 -17 -18 -19 -20 27 37 23 -22 29 28 25 $  >*Scirtothrips dorsalis* East Asia 1 (SDEA1)  1 10 2 3 5 12 39 9 4 8 32 27 21 22 13 15 33 24 -38 35 36 26 34 11 16 30 6 7 14 31 -17 -18 -19 -20 37 23 29 28 25 $  >*Scirtothrips dorsalis* South Asia 1 (SDSA1)  1 10 2 3 5 12 9 4 8 32 27 21 22 13 15 33 24 38 35 36 26 34 11 -16 30 6 7 14 31 -17 39 -18 -19 -20 29 28 25 37 23 40 $  >*Frankliniella intonsa* (FI)  1 10 2 3 5 12 9 4 8 32 21 39 24 33 40 -22 -38 35 36 26 34 11 16 30 6 7 13 15 14 27 31 -17 -18 -19 -20 37 23 29 28 25 $  >*Frankliniella occidentalis* (FO)  1 10 2 3 5 12 9 4 8 39 21 33 32 24 40 -22 -38 35 36 26 34 11 16 30 6 7 13 15 14 27 31 -17 -18 -19 -20 37 23 29 28 25 $  >*Thrips imaginis* (TI)  1 10 2 3 9 4 8 12 21 13 15 39 -22 32 24 -38 35 36 26 34 11 16 30 6 7 33 25 5 -14 27 31 -17 -18 -19 -20 37 23 29 28 $  >A1  1 10 2 3 5 12 9 4 8 40 21 22 13 15 33 32 24 -38 35 36 26 34 11 16 30 6 7 14 31 -17 -18 -19 -20 27 37 23 29 28 25 $  >A2  1 10 2 3 5 12 9 4 8 40 21 22 13 15 33 32 24 -38 35 36 26 34 11 16 30 6 7 14 31 -17 -18 -19 -20 27 37 23 29 28 25 $  >A3  1 10 2 3 5 12 40 9 4 8 32 27 21 22 13 15 33 24 -38 35 36 26 34 11 16 30 6 7 14 31 -17 -18 -19 -20 37 23 29 28 25 $  >A4  1 10 2 3 5 12 40 9 4 8 32 27 21 22 13 15 33 24 -38 35 36 26 34 11 16 30 6 7 14 31 -17 -18 -19 -20 37 23 29 28 25 $  >A5  1 10 2 3 5 12 9 4 8 40 21 22 13 15 33 32 24 -38 35 36 26 34 11 16 30 6 7 14 27 31 -17 -18 -19 -20 37 23 29 28 25 $  >A6  -25 -28 -29 -23 -37 20 19 18 17 -31 -27 -14 -15 -13 -7 -6 -30 -16 -11 -34 -26 -36 -35 38 22 -41 -24 -32 -33 -21 -40 -8 -4 -9 -12 -5 -3 -2 -10 -1 $ |
| --- |
